# Combined Phacoendoscopic Cyclophotocoagulation versus Combined Phacotrabeculectomy in the Management of Coexisting Cataract and Glaucoma: A Comparative Study

**DOI:** 10.1155/2018/5149154

**Published:** 2018-12-16

**Authors:** Charles Sing Lok Lau, Jeffrey Chi Wang Chan, Sophia Fei So, Orlando Chia Chieh Chan, Kenneth Kai Wang Li

**Affiliations:** ^1^Department of Ophthalmology, United Christian Hospital, Kowloon, Hong Kong; ^2^Department of Ophthalmology, Tseung Kwan O Hospital, Tseung Kwan O, Hong Kong

## Abstract

**Purpose:**

To compare the surgical outcome of combined phacoemulsification and endoscopic cyclophotocoagulation (phacoECP) versus combined phacoemulsification and mitomycin C-augmented trabeculectomy (phacoTbx) in patients with coexisting glaucoma and visually significant cataract.

**Methods:**

A retrospective review of 89 eyes of 89 patients who received phacoECP (*N*=49) and phacoTbx (*N*=40) was carried out at a tertiary eye center in Hong Kong. The minimum follow-up period was 6 months. Criterion of success was reduction of IOP at least 30% or absolute IOP of 15 mmHg or below without (complete success) or with (qualified success) antiglaucomatous medication.

**Results:**

PhacoTbx had more reduction of antiglaucomatous medication (4 vs 1, *P* < 0.001). At postoperative year one, there was more IOP reduction for phacoTbx than phacoECP (8 mmHg vs 3 mmHg, *P*=0.012). The one-year complete success rate was also higher for phacoTbx (46.2% vs 8.2%, *P* < 0.001), while qualified success was comparable between the 2 groups (74.4% vs 73.5%, *P*=0.925). Operation time was shorter for phacoECP (37 vs 73 minutes, *P* < 0.001). The number of postoperative follow-up visits was less (6 vs 11.5, *P* < 0.001) for phacoECP. Additional surgical procedures were more common in phacoTbx (55% vs 0%, *P* < 0.001). There was no postoperative cystoid macula edema, hypotony, or endophthalmitis reported in both groups.

**Conclusions:**

PhacoECP is significantly less effective than phacoTbx in reduction of both IOP and number of antiglaucomatous medications for patients with medically uncontrolled glaucoma and cataract. Its complete success rate is also significantly lower than that of phacoTbx. With its comparable qualified success, shorter operation time, less number of postoperative visits, and secondary surgical intervention, phacoECP may still have a role in very selected cases.

## 1. Introduction

Combined phacoemulsification, intraocular lens (IOL) implantation, and trabeculectomy (phacoTbx) is the most popular combined procedure for the management of coexisting glaucoma and cataract. With the adjunctive use of mitomycin C, phacoTbx has been shown to be effective to lower intraocular pressure (IOP) by 6.5 to 10.7 mmHg [1]. However, cases may be subjected to increase in postoperative inflammation, bleb-related complication, or hypotony, causing difficult postoperative management.

Endoscopic cyclophotocoagulation (ECP) reduces aqueous production through controlled ablation of ciliary body processes using laser photocoagulation under direct visualization. It has been proved to be effective in glaucoma management with less postoperative inflammation compared to phacoTbx [2]. When combined with cataract extraction (phacoECP), studies have shown that phacoECP could reduce IOP by 17.6% to 46.9%, and the percentage of antiglaucomatous medication use could be reduced by 21.9% to 79.7% [3]. The present study aims to compare the surgical outcome of phacoECP and phacoTbx in a retrospective manner.

## 2. Materials and Methods

### 2.1. Study Design

This is a retrospective case-control study. Approval was obtained from the local institutional review board (Reference no. KC/KE-15-0049/ER-1), and the study was conducted in accordance with the principles of Declaration of Helsinki.

### 2.2. Eligibility Criteria

All consecutive cases of coexisting glaucoma and visually significant cataract that underwent phacoTbx or phacoECP, during January 2012 to May 2014, in the ophthalmic services of the Kowloon East Cluster of Hospital Authority of Hong Kong were retrospectively included in the study. The minimum follow-up period was 6 months. Patients younger than 18 years of age were excluded from the study.

### 2.3. Data Collection

Patients' demographics, diagnosis and types of glaucoma, pretreatment information (including preoperative number of antiglaucomatous medications, IOP by Goldmann tonometry, and Snellen visual acuity (VA)), intraoperative details (including types of operation, operation time, and complications), postoperative details (including anterior chamber inflammation, complications, and IOP on day 1, 1 month, 3 months, 6 months, and 1 year), VA (1 year), number of antiglaucomatous medications at 1 year, number of outpatient clinic visits, and any additional related surgical interventions within the postoperative 6-month period) are collected. Post-op anterior chamber inflammation was categorized into group 1: anterior chamber cells less than or equal to 2+, group 2: anterior chamber cells more than 3+, and group 3: presence of fibrinous reaction.

### 2.4. Surgical Technique

All procedures were performed by 2 glaucoma specialists (OC and SS). All cases were given regional anesthesia. After completion of phacoemulsification and cortical cleanup but before intraocular lens implantation, ECP (Endo Optiks E2; Beaver-Visitec, Waltham, MA, USA) was performed [4]. The viscoelastic substance was injected to open the sulcus space. Laser power ranges from 300 mW to 500 mW with continuous wave duration. Choice of coverage of the laser treatment (180–240° or 300–360° of ciliary processes) was based on the patient's preoperative baseline IOP; additional corneal incision would be created 180° away from the initial phacoemulsification wound in cases where treatment involved 300–360°. After ECP, IOL was implanted followed by removal of all ophthalmic viscosurgical devices. Wounds would be closed with either stromal hydration or with 10-O suture at the end of the procedure.

For phacoTbx, the fornix-based conjunctival flap approach was adopted in all cases. After creation of conjunctival flap and partial-thickness scleral flap, sponges soaked with mitomycin C (0.4 mg/mL) were applied under the conjunctival and scleral flaps for 3 minutes before thorough irrigation. Then, a clear corneal phacoemulsification and IOL implantation were performed. After closure of the corneal wound with the 10-O nylon suture, a sclerostomy was created under the scleral flap using a Kelly Descemet punch, followed by a surgical peripheral iridectomy. The scleral flap and conjunctival flap were closed with interrupted 10-O and 8-O nylon sutures, respectively.

For both surgeries, all eyes were patched with dexamethasone-neomycin-polymyxin B ointment for 1 day after operation and were prescribed with either combined dexamethasone 0.1% + chloramphenicol 0.5% eye drops 6–8 times per day or levofloxacin 0.5% eye drops 4 times per day with prednisolone acetate 1% eye drops 6–8 times per day, and the eye drops would be tapered down gradually from 2 to 4 weeks after operation based on the clinical progress. In cases of phacoECP, nepafenac eye drops 3 times per day were prescribed. In the phacoTbx group, all preoperative antiglaucomatous medications were stopped after surgery, while in the phacoECP group, the medications were continued in the initial postoperative period and subsequently tapered down according to the patient's IOP.

### 2.5. Surgical Success

Qualified success was defined as IOP below or equal to 15 mmHg or IOP drop at least 30% with or without antiglaucomatous medication, and complete success was defined as IOP below or equal to 15 mmHg or IOP drop at least 30% without any use of antiglaucomatous medication.

### 2.6. Statistical Analysis

All statistical analyses were performed with SPSS software (version 22; IBM). Normality of the distribution of each datum was assessed with the Kolmogorov–Smirnov statistic. Since the parameters in our dataset did not follow normal distribution, descriptive statistic would be presented in median and interquartile range. For cases with both eyes operated, only one eye is chosen for analysis by randomization with Excel 2016 (Microsoft). Pre- and postoperative changes in IOP, medication, and VA were analyzed with the Friedman test and Wilcoxon signed-ranks test. Intergroup comparisons of operative time, number of postoperative visits, additional surgical procedures, reduction of medication, and reduction of IOP were analyzed with the Mann–Whitney *U*-test. Postoperative inflammation, complications, and surgical success rate between groups were analyzed with the chi-square test or Fisher exact test. Level of significance is defined as *P* < 0.05.

Further analysis was performed to compare results between phacoECP and phacoTbx in primary open-angle glaucoma (POAG) and chronic angle-closure glaucoma (CACG)/primary angle-closure glaucoma (PACG) using the Mann–Whitney test (for IOP/medication reduction), Fisher exact test, and chi-square test (for success rate); also, results are compared for different subgroups undergoing phacoECP by the Kruskal–Wallis test. Uveitic glaucoma and neovascular glaucoma were excluded in the subgroup analysis because only phacoTbx was performed for those cases in our series.

## 3. Results

A total of 89 eyes of 89 patients were recruited for this study (there were 16 patients with both eyes operated, and only 1 eye was randomly selected for analysis). 49 eyes received phacoECP and 40 eyes received phacoTbx; the choice of which glaucoma surgery to perform depends on the patient's IOP control, surgeon's preference, and patient's preference after thorough discussion with the patient on available choices. One patient in the phacoTbx group died of lung cancer 10 months after operation; thus, there were no 1-year postoperative data obtained for this case. One case in the phacoECP group and 2 cases in the phacoTbx group received trabeculectomy before. Parameters including age and VA were comparable between the two groups. Median preoperative IOP and number of antiglaucomatous medications were significantly higher for the phacoTbx group (20.5 mmHg, 4 meds) as compared to the phacoECP group (18 mmHg, 4 meds; *P*=0.003 and 0.008, respectively) (Table 1).

Both phacoECP and phacoTbx groups showed significant reduction of IOP across all time points (*P* < 0.001, Friedman test). Subsequent post hoc comparison found significant reduction in IOP for day 1, 1 month, 3 months, 6 months, and 1 year after operation as compared to preoperative IOP (all with *P* < 0.001, by the Wilcoxon signed-ranks test with Bonferroni adjustment). At postoperative year one, IOP was reduced by 3 mmHg (13.6% IOP reduction) for phacoECP (*P* < 0.001) and 8 mmHg (33% IOP reduction) for phacoTbx (*P* < 0.001), with a significant difference between the 2 groups (*P*=0.012).

At 1 year after operation, there was reduction of 1 antiglaucomatous medication for the phacoECP group (*P* < 0.001) and 4 antiglaucomatous medications for the phacoTbx group (*P* < 0.001). The difference between two groups was also statistically significant (*P* < 0.001). Qualified success in 1 year was achieved in 36/49 (73.5%) and 29/39 (74.4%) of the phacoECP group and phacoTbx group, respectively (*P*=0.925). Complete success was achieved in 4/49 (8.2%) of the phacoECP group and 18/39 (46.2%) of the phacoTbx group, with a significant difference between the 2 groups (*P* < 0.001).

For phacoECP, it has significantly shorter operation time (37 vs 73 min, *P* < 0.001) and less follow-up visits in initial 6 months after operation (6 vs 11.5 visits, *P* < 0.001), and there were less cases requiring additional surgical procedures to enhance IOP control in the initial 6 months after operation (0/49 vs 22/40, *P* < 0.001) when compared to phacoTbx (subconjunctival 5-fluorouracil injection only in 7/40 and bleb needling with subconjunctival 5-fluorouracil injection in 15/40).

Postoperative anterior chamber inflammation at day 1 and 1 month was comparable between the two groups (*P*=0.18 and *P*=0.39, respectively). VA improvement (in logMAR) was 0.176 for phacoECP (*P* < 0.001) and 0.125 for phacoTbx (*P*=0.001), but the difference was insignificant (*P*=0.943).

Subgroup analysis was performed comparing the 3 types of glaucoma (POAG, CACG/PACG, and NTG), and the results are summarized in Tables 2 and 3. Among the 3 types of glaucoma undergoing phacoECP, there was no statistically significant difference in success rate, IOP reduction, and antiglaucomatous medication reduction. Comparing success of phacoECP and phacoTbx in POAG and CACG/PACG, it was found that the rate of complete success for phacoTbx in POAG is better than that for phacoECP (*P*=0.002), and there was more antiglaucomatous medications reduction for POAG and CACG/PACG in phacoTbx (*P*=0.001 and *P*=0.023, respectively).

Further analysis was done to study the effect of phacoECP on mild-to-moderate glaucoma (defined as cases with the vertical cup-to-disc ratio less than 0.8 with IOP controlled medically with not more than 3 medications and minimal glaucomatous field loss) and severe glaucoma; it showed that the complete success rate was 1/5 (20%) and 3/44 (6.8%), respectively (*P*=0.31), and the qualified success rate was 3/5 (60%) and 33/44 (75%), respectively(*P*=0.47).

For the phacoECP group, no intraoperative complication was encountered; there was one case of post-op retained cortical matter which resolved spontaneously with conservative treatment. For the phacoTbx group, there were 3 cases of intraoperative self-limiting iris bleed and 1 case of postoperative corneal wound leak requiring wound repair. The complication rate was comparable between the two groups (phacoECP 2.0% and phacoTbx 10%, *P*=0.170). No cases of hypotony, choroidal detachment, cystoid macula edema, or endophthalmitis after operation were encountered. Two cases of phacoTbx required additional glaucoma operation (glaucoma drainage device in both cases) for better IOP control (operated 7 and 10 months after phacoTbx) where 1 case of phacoECP required additional glaucoma operation (trabeculectomy with MMC) 8 months after phacoECP.

## 4. Discussion

There are limited data studying the difference in effectiveness between combined phacoECP and phacoTbx on the treatment of glaucoma, especially in Asian eyes. It was suggested that, with racial difference, in particular the difference in the pigmentation in the ciliary epithelium, there is a difference in the energy delivery to the ciliary epithelium by ECP [5]. In the present study, we adopted a stringent criterion for complete success (15 mmHg or below or IOP reduction at least 30%) similar to the previous study by Morales et al. [6]. This is because the majority of our cases had advanced glaucomatous cupping (cup-to-disc ratio more than 0.7: 44/49 in the phacoECP group and 31/40 in the phacoTbx group), and therefore, they required a much lower target IOP to prevent progressive visual field loss.

Our results showed that the complete success rate of phacoECP is lower than that of phacoTbx, which is consistent with the previous study [7]. This could be explained by the results of a histopathology study published by Lin et al. [8] which showed that there is some reperfusion of the ciliary body 1 month after ECP. On the contrary, phacoECP has the advantage of significantly shorter operation time with easier postoperative management to glaucoma patients. Together with its comparable qualified success rate to phacoTbx, phacoECP may still be beneficial to patients who have difficulties with frequent follow-up (e.g., patients with mobility difficulties) and also for patients who cannot tolerate long operations because of systemic morbidities (e.g., patients with back or airway problems).

Another advantage of phacoECP is the avoidance of life-long risks of bleb-related complications such as bleb leak and bleb-related infections, which was estimated to be 2.0% at 10 years by Kim et al. [9].

PhacoECP may also have a role in patients who are poor candidates for phacoTbx such as those with a history of multiple ocular surgeries with scarred conjunctiva, high risk of systemic morbidities when withholding antiplatelet or anticoagulant in the perioperative period, or poor outcome of previous filtration surgery in the same eye or fellow eye due to excessive scarring and wound healing at the filtration site.

Although phacoECP has been advocated for mild-to-moderate glaucoma in previous studies [10], our study has also shown the beneficial effect of phacoECP on advanced glaucoma, and the overall success rate was comparable with that in another study by Morales et al., which studied the effect of phacoECP on advanced glaucoma (absolute success: 11.9%; qualified success: 72.3%) [6]. Our study failed to identify any significant difference in the outcome of phacoECP between mild-to-moderate glaucoma and severe glaucoma, but it could be related to the small sample size of the mild-to-moderate cases (*N*=5). Other than phacoECP's less favourable IOP control, the reduction in the number of antiglaucomatous medications was also found to be significantly less in phacoECP compared to phacoTbx (1 vs 4 meds reduction). Therefore, patients with poor drug compliance may not be good candidates for phacoECP.

Our study suffers from the inherent limitations of case-control studies. Other than no randomization and inclusion of cases with heterogeneous diagnoses, the baseline IOP and numbers of antiglaucomatous medications are higher for the phacoTbx group. Owing to the quicker IOP lowering effect of phacoTbx which was shown previously [7], phacoTbx was the preferred choice of surgery for cases with very high preoperative IOP and those with poor IOP control even on maximal antiglaucomatous medication; it also leads to uneven distribution of NTG and other types of glaucoma between the two groups. Thus, the further randomized control trial is warranted in the future to compare the effect of the two methods for glaucoma patients.

In summary, our study showed that phacoECP is significantly less effective than phacoTbx in terms of complete success rate, IOP reduction, and the reduction of the number of antiglaucomatous medications. Nevertheless, phacoECP with comparable qualified success to phacoTbx, shorter operation time, less postoperative follow-up visits, and the avoidance of bleb-related complications could still have a role for the management of medically uncontrolled glaucoma in very selected cases.

## Figures and Tables

**Table 1:** Demographic data, diagnosis, and baseline parameters for patients receiving phacoECP or phacoTbx.

Parameters	*N*	
	PhacoECP	PhacoTbx
Age (median (interquartile range))	74 (15)	69.5 (12)
Gender		
Male	34	23
Female	15	17
Baseline IOP (median)^*∗*^	18	20.5
Baseline medications (median)^$^	4	4
Baseline VA (logMAR)	0.523	0.523
Diagnosis		
POAG	31	22
CACG/PACG	9	12
NTG	9	0
Others^#^	0	6

^*∗*^ *P*=0.003. ^$^*P*=0.008. ^#^1 neovascular glaucoma and 5 uveitic glaucomas. POAG: primary open-angle glaucoma; CACG: chronic angle-closure glaucoma; PACG: primary angle-closure glaucoma; NTG: normal-tension glaucoma.

**Table 2:** Comparison of results of different subgroups in phacoECP.

Subgroup	*N*	IOP reduction (mmHg)	Med reduction	Qualified success, *N* (%)	Complete success, *N* (%)
POAG	31	3	1	22 (71)	1 (3.2)
CACG/PACG	9	3	2	6 (66.7)	2 (22.2)
NTG	9	2	1	8 (88.9)	1 (11.1)
*P* value^*∗*^		0.718	0.054	0.494	0.175

^*∗*^Intergroup comparison by the Kruskal–Wallis test.

**Table 3:** Comparison of results between phacoECP and phacoTbx in different subgroups.

Subgroup	Number of cases		Qualified success, *N* (%)		*P* value^*∗*^	Complete success, *N* (%)		*P* value^*∗*^	IOP reduction (mmHg)		*P* value^#^	Med reduction		*P* value^#^
	PhacoECP	PhacoTbx	PhacoECP	PhacoTbx		PhacoECP	PhacoTbx		PhacoECP	PhacoTbx		PhacoECP	PhacoTbx	
POAG	31	22 ^	22 (71.0)	16 (76.2)	0.677	1 (3.2)	8 (38.1)	**0.002**	3	6	0.137	1	4	**0.001**
CACG/PACG	9	12	6 (66.7)	10 (83.3)	0.611	2 (22.2)	7 (58.3)	0.184	3	8	0.144	2	3	**0.019**

^*∗*^Analyzed by chi-square test/Fisher exact test. ^#^Analyzed by Mann–Whitney test. ^ 1 case lost to follow-up after 6 months.

## Data Availability

The data used to support the findings of this study are included within the article.
